# Characterizing the Clinical and Molecular Profile of SETD2-Mutated Lung Adenocarcinoma

**DOI:** 10.3390/cancers17213540

**Published:** 2025-11-01

**Authors:** Omar Bushara, David Devaro, Shawn S. Ahn, Jordan Dourlain, Mohammad S. Farooq, Alessandro Brunetti, Simon Chen, David Feldser, John C. Kucharczuk, Sunil Singhal

**Affiliations:** 1 Department of Surgery, Hospital of the University of Pennsylvania, Philadelphia, PA 19104, USA; 2 Department of Pathology and Laboratory Medicine, Hospital of the University of Pennsylvania, Philadelphia, PA 19104, USA; 3 Department of Cancer Biology, Perelman School of Medicine, University of Pennsylvania, Philadelphia, PA 19104, USA

**Keywords:** non-small cell lung cancer, adenocarcinoma, *SETD2*, next generation sequencing

## Abstract

**Simple Summary:**

*SETD2* is an understudied gene that encodes for a histone methyltransferase implicated in lung cancer tumorigenesis. The goal of this study is to elucidate the characteristics and prognosis of *SETD2*-mutated lung adenocarcinoma. With the largest cohort to date, we demonstrate that *SETD2*-mutated lung adenocarcinoma presents at significantly earlier stages, has a unique molecular profile compared to non-mutated tumors, and trends towards improved RFS in early-stage tumors. Further study is warranted to immunopathologic basis of these findings.

**Abstract:**

**Introduction**: *SETD2* is an understudied gene that encodes for a histone methyltransferase implicated in lung cancer tumorigenesis and is found in up to 10% of all non-small cell lung cancer. With the largest cohort to date, we aim to elucidate the clinicopathologic characteristics and prognosis of *SETD2*-mutated lung adenocarcinoma. **Methods**: We obtained molecular genetics reports of lung cancer seen between 1 January 2015 and 4 January 2024. We identified all *SETD2*-mutated cancer and identified 500 consecutive cases prior to 4 January 2020 as a control group. Non-recurrent adenocarcinomas were included. Patient and tumor characteristics and recurrence-free survival (RFS) were compared. Fisher’s exact, Wilcoxon rank sum, and log-rank tests were used when appropriate. Kaplan–Meier plots and Cox proportional hazards models were used to analyze recurrence-free survival. **Results**: A total of 67 *SETD2*-mutated lung and 174 non-*SETD2*-mutated lung adenocarcinomas met inclusion criteria. *SETD2*-mutated tumors presented at earlier stages (55.2% vs. 17.8% stage I, 11.9% vs. 48.3% stage IV, *p* < 0.001). *SETD2*-mutated adenocarcinoma had a higher number of genetic mutations (median: 11 IQR: [8–15] vs. 7 [5–10], *p* < 0.001). In a univariable Cox analysis, *SETD2* mutation was associated with improved RFS (HR 0.53 95% CI: [0.33–0.85], *p* = 0.008). In a covariate-adjusted model, *SETD2* mutation trended towards improved RFS (0.71 [0.43–1.18], *p* = 0.10). **Conclusions**: These data suggest *SETD2*-mutated lung adenocarcinoma presents at significantly earlier stages, has a unique molecular profile compared to non-mutated tumors, and trends towards improved RFS in early-stage tumors. Future study is warranted on both patient outcomes and immunopathologic characteristics of *SETD2*-mutated lung adenocarcinoma.

## 1. Introduction

Lung cancer remains the most common cause of cancer-related death in the United States [1]. Non-small cell lung cancer represents over 80% of all lung cancer, with adenocarcinoma being the most common subtype [2]. Investigation into driver mutations has yielded targeted therapies, such as those for *EGFR* mutations and *ALK* and *ROS1* fusions, which have in turn improved patient outcomes [3]. The routine use of next-generation sequencing has made the molecular profiles of lung tumors more readily studied and facilitates further research on potentially significant mutations, such as SETD2 [4].

*SETD2* is frequently mutated in multiple cancer types and encodes the histone 3 lysine 36 (H3K36) trimethyltransferase. *SETD2* is physiologically involved in chromatin regulation [5,6]. Additionally, more recent research has identified a role of SETD2 in cytoskeletal and cellular migratory function [7,8,9]. The loss of H3K36 methylation is associated with defects in alternative splicing and DNA repair [9,10]. There are no known risk factors for *SETD2* mutation. Although understudied, *SETD2* inactivation has been implicated in the tumorigenesis of a wide variety of cancers, including lung adenocarcinoma [9].

In lung adenocarcinoma, *SETD2* inactivation has been validated to accelerate *KRAS*-driven tumor growth in murine models [11,12,13]. Despite the implication of *SETD2* inactivation in the growth of lung cancer, several studies have shown it is a potentially favorable prognostic factor. *SETD2*-mutated cancers may be more sensitive to radio- and immunotherapy [14,15,16]. Although a promising biomarker, there is a dearth of pre-clinical research in this area, and the existing clinical studies have small sample sizes. Additionally, the paradoxical finding that pre-clinical data demonstrates pro-tumorigenic effects however clinical data thus far has consistently demonstrated more favorable outcomes underscores the need for further investigation into SETD2-mutated lung cancer.

With the largest cohort to date, we describe the clinical characteristics and molecular profiles of SETD2-mutated lung adenocarcinoma from a large cohort of patients at a single institution. We also aim to elucidate the utility of *SETD2* mutations for prognostication by comparing recurrence-free survival (RFS) in patients with and without this mutation.

## 2. Methods

In this study we obtained molecular genetics reports (221 gene panel) for lung cancer seen at our institution between 1 January 2015 and 4 January 2024 in patients over the age of 18. We identified all cases of *SETD2*-mutated lung cancer, 229 in total. We then identified a convenience sample of the 500 consecutive cases of lung cancer immediately prior to 4 January 2020 to serve as a control group. All recurrent cases, small cell lung cancer, and pleural malignancies were excluded from both groups. Based on a primary review of the pathology reports of the remaining patients, we only included adenocarcinomas for analysis.

Clinical tumor samples were processed for NGS at the Center for Personalized Diagnostics (CPD) laboratory, a CLIA-certified and CAP-accredited cancer genomics laboratory associated with the Hospital of the University of Pennsylvania. Each specimen was sequenced on one of the three NGS panels described below in Supplemental Methods.

Clinical and pathologic variables were abstracted via chart review. Patient covariates included age, BMI, gender, a history of tobacco smoking, COPD, asthma, and asbestos exposure. TNM staging was noted at diagnosis and was based on standard AJCC guidelines. PD-L1 positivity was defined as >10% expression noted in the surgical pathology report. Patients with incomplete data or who were lost to follow-up were excluded from both groups.

The primary outcome measure was recurrence-free survival. As a subgroup comparison, we grouped those presenting as stage I or II as “early-stage” and those at stages III or IV as “advanced-stage.” The sample size did not facilitate further stratification, and as such this grouping was chosen.

Standard descriptive statistics were used. Fisher’s exact test and Wilcoxon rank sum test compared categorical variables and continuous variables, respectively. Bonferroni correction was used when comparing mutation frequencies. A total of 30 mutations were analyzed, with the threshold for significance in this comparison at *p* < 0.0016 (0.05/30). This was done in order to avoid spurious significant associations given the high number of serial tests.

Kaplan–Meier (KM) analysis was conducted for overall survival stratified by the presence or absence of *SETD2* mutation. Unadjusted recurrence-free survival was compared using the log-rank tests. We utilized a parsimonious Cox proportional hazards model to analyze the association between *SETD2* mutation and adjusted recurrence-free survival. Using individual Cox regressions, we identified covariates with *p* < 0.2 to include in our final multivariable model. All variables with *p* < 0.2 included *SETD2* mutation, age, diabetes, COPD, and advanced stage at presentation. All analyses were performed using Stata 18 (StataCorp LP, College Station, TX, USA).

This study also attempted to elucidate the significance of adjuvant immunotherapy in *SETD2*-mutated tumors in a post hoc analysis. However, due to the sample size this was not meaningful. As a sensitivity analysis, the inclusion of receipt of immunotherapy did not change other effect sizes, significance, or directionality of our study.

This study was approved by the University of Pennsylvania Institutional Review Board with protocol code 857662 on 8 January 2025.

## 3. Results

Of the 3078 cases of lung cancer diagnosed between 1 January 2015 and 4 January 2024, 229 (7.4%) were *SETD2*-mutated. Of these, 67 *SETD2*-mutated lung adenocarcinomas met inclusion criteria. Of 500 consecutive patients seen between October 2018 and 4 January 2020, 174 non-*SETD2*-mutated lung adenocarcinomas met inclusion criteria.

The stage at clinical presentation was found to differ between groups, with *SETD2*-mutated tumors presenting at earlier stages (55.2% vs. 17.8% for stage I and 11.9% vs. 48.3% for stage IV, *p* < 0.001). *SETD2*-mutated adenocarcinoma was also found to have a higher number of mutated genes (median: 11 IQR: [9–16] vs. 7 [5–10], *p* < 0.001) (Table 1).

*SETD2*-mutated tumors demonstrated a unique molecular profile, with higher rates of TMB, mutations in *SLIT2*, *MED12*, and *KMT2D* and lower rates of mutation in *EGFR*, *MET* and *ERBB2* (all *p* < 0.001). *KRAS* mutation rate did not differ between groups, although was nominally higher in *SETD2*-mutated tumors (47.7% vs. 33.9%) (Table 2).

A total of 93 *SETD2* mutations were represented by 67 patients, with 21 having multiple mutations. The typical mutations identified were missense (35, 37.6%), followed by frameshift (27, 29.0%) (Supplemental Table S1). There were 4 mutations with uncertain protein effects and 89 single-instance mutations.

**Figure 1:**
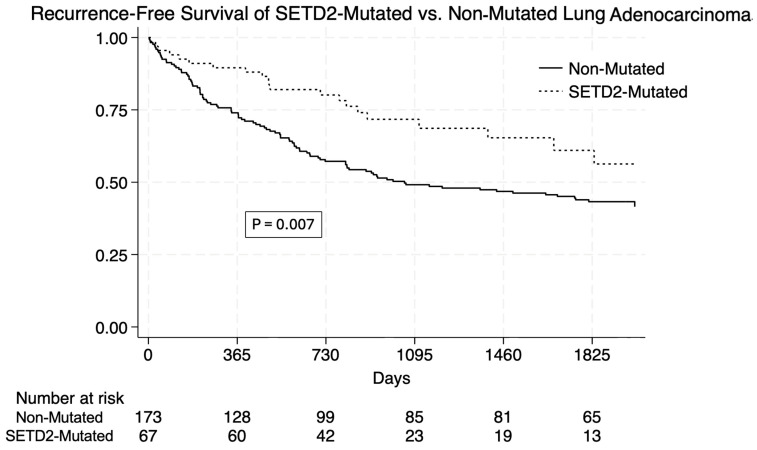
Kaplan–Meier plots of recurrence-free survival in patients with and without SETD2 mutation. Recurrence-free survival was significantly improved in SETD2-mutated patients, with log-rank test showing *p* = 0.007.

**Figure 2:**
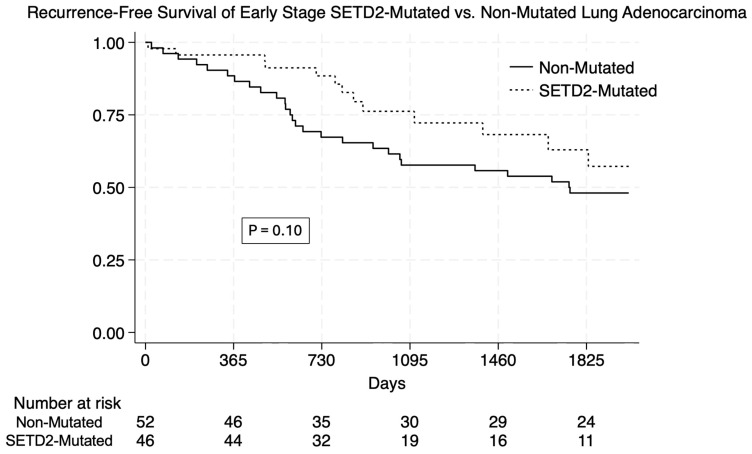
Kaplan–Meier plots of recurrence-free survival in patients with and without SETD2 mutation. In patients with early-stage (stages I and II) tumors, there was a trend towards improved recurrence-free survival, with long-rank test showing *p* = 0.10.

*SETD2*-mutated lung adenocarcinoma had a higher unadjusted one-, three-, and five-year RFS (Table 3). The overall RFS was improved in *SETD2*-mutated tumors (*p* = 0.007 via log-rank test). (Figure 1) In a stratified analysis of early-stage (I or II) tumors, *SETD2* mutation trended towards significantly improved survival (*p* = 0.10) (Figure 2). There was no difference in RFS in advanced-stage tumors (*p* = 0.27).

Finally, in a univariable Cox analysis, *SETD2* mutation was associated with improved RFS (HR 0.53 95% CI: [0.33–0.85], *p* = 0.008). Separate univariate analyses identified age (*p* = 0.08), diabetes (0.13), COPD, and an advanced (III or IV) stage (0.01) as *p* < 0.2. These were included in a multivariable model. In this covariate-adjusted model, *SETD2* mutation trended towards improved RFS (0.71 [0.43–1.18], *p* = 0.10) and an advanced stage was found to be associated with worse RFS (1.25 [1.06–1.47], *p* = 0.02).

Only 14 (20.8%) patients with SETD2 mutations received any type of immunotherapy compared to 77 (44%) of non-mutated patients, likely due to differing stages at presentation and years of diagnosis and treatment. As such, we were unable to conduct a meaningful analysis. A sensitivity analysis was performed, including the receipt of immunotherapy in the multivariable model, which yielded *p* = 0.18 and did not change the effect sizes or significance of other variables.

## 4. Discussion

The literature investigating *SETD2* mutation thus far is limited to pre-clinical studies and small clinical cohorts [14,15,16]. Herein, with the largest clinical cohort to date, we demonstrate that *SETD2*-mutated lung adenocarcinoma presents at a significantly earlier stage and trends towards improved recurrence-free survival in early-stage tumors. Secondarily, we define the clinical and molecular characteristics of *SETD2*-mutated lung adenocarcinoma and demonstrate that *SETD2*-mutated tumors have a unique genetic profile. Finally, given the dearth of investigations into SETD2-mutated lung adenocarcinoma, we hope this manuscript is hypothesis-generating and lays the foundation for future research.

These data suggest that *SETD2*-mutated lung adenocarcinoma may be associated with a more favorable tumor phenotype. First, the *SETD2* mutation is associated with an earlier stage at presentation compared to their non-mutated counterparts. This may suggest that these adenocarcinomas are less aggressive and grow more slowly and thus are more likely to present in stages I or II. The relationship between *SETD2* mutation and nodal or distant metastasis is not known; however, tumors with phenotypes associated with *SETD2* mutations may be less likely to metastasize. *SETD2* has been shown to methylate actin and tubulin in a variety of contexts, and this cytoskeletal and mitotic regulation may mediate the likelihood of cellular metastasis [7,8]. The molecular and biologic basis of the association of *SETD2* mutation and an earlier stage at presentation warrants future investigation. More specifically, the link between cytoskeletal regulation and metastatic potential is an area of investigation.

Further, in early-stage tumors, *SETD2* mutation was associated with a trend towards significantly improved recurrence-free survival, potentially demonstrating a favorable prognostic value. This may be due to improved sensitivity to available treatments—*SETD2* mutation in lung cancer has been associated with an improved response to radio- and immunotherapy [14,15,16]. This also may be related to the risk of nodal and distant metastasis as described above [7,8]; however, other mechanisms may underly this observation. The lack of significance in advanced-stage tumors may underscore the importance of the early diagnosis and treatment of lung cancer. Patients who are already at later stages may have a comparable mortality risk. Finally, these findings were present without significant differences in patient characteristics or PD-L1 expression, possibly also suggesting that tumor biology underlies the difference in outcomes, as other included covariates do not differ.

Overall, our primary findings are concordant with the current literature on lung cancer. *SETD2* has been demonstrated to be associated with favorable treatment responses [14,15,16]. This is the first and largest study to show that these tumors present at significantly earlier stages. Interestingly, the potential favorable prognostic value of the *SETD2* mutation in lung cancer differs from that described in clinical studies of other cancers. For example, *SETD2* is suggested to be associated with a more aggressive tumor phenotype in GISTs and pancreatic cancer [17,18]. This may suggest differing roles in each cancer context, which may explain the diverging associations. However, pre-clinical models of lung cancer suggest that *SETD2*-mutated tumors are associated with rapid tumor growth, but a relatively lower likelihood of metastasis compared to other pre-clinical lung cancer models [11]. This is in line with our clinical observations and may explain the lower stage at presentation and the trend towards improved RFS.

In addition to the clinical characteristics of *SETD2*-mutated tumors, we also characterized their molecular profiles. We demonstrated that *SETD2*-mutated tumors had a higher number of total mutations than their counterparts. Further, of the genes that had at least a 10% mutation rate in the *SETD2* mutant cohort, the co-mutation rate for seven genes differed significantly compared to the non *SETD2*-mutated group. *SETD2*-mutated tumors had higher TMB and co-mutation rates of *SLIT2*, *MED12*, and *KMT2D*. However, *SETD2* mutation had lower rates of *EGFR*, *MET,* and *ERBB2* mutations. Although *SETD2* has been shown to accelerate KRAS-mediated tumorigenesis, the frequency of *KRAS* mutation did not significantly differ between groups [14]. Notably, *EGFR*, *MET*, and *ERBB2* encode for tyrosine kinase receptors [19,20], possibly suggesting that oncogenic pathways involving these mutations are not compatible with *SETD2* mutations due to a functional necessity. This is an area of active investigation and may identify downstream processes regulated by *EGFR* that require intact *SETD2*.

Finally, this is also one of the few studies to attempt to characterize the types and locations found in *SETD2* mutation. We found that the majority are missense or frameshift mutations, and that there are no mutation hotspots within the *SETD2* gene. This is in line with the available literature on other cancer types, which also demonstrates that the majority are missense or frameshift mutations and a distinct lack of mutation hotspots in *SETD2* [21].

This study, although concordant with the available clinical literature, is limited to clinical observation and thus does not conclusively link *SETD2* biology with outcomes. Of specific importance, this study is not able to address the differing pre-clinical and clinical findings that *SETD2* is associated with tumor growth in *KRAS*-driven adenocarcinoma, while also being associated with more favorable outcomes. This may be explained by SETD2-mutated tumors being more likely to be mass-forming as they progress. These may be reasoned to be more likely to be symptomatic and be more likely to be detected on imaging. SETD2 mutation may also be associated with tumor growth but not metastasis, which may also allow time for detection at node-negative, non-metastatic earlier stages of disease. Additionally, there are potential confounders that may influence our findings. First, there may be a selection bias in referral for NGS, especially in earlier years in our cohort. However, the full cohort was composed of patients with NGS testing, so this bias may be equally represented in both the *SETD2*-mutated group and the control. Additionally, late-stage *SETD2*-mutated cancers may not be fully represented in this study, and thus a survival bias may be introduced. Although our study identifies a potentially more favorable cancer phenotype, further translational investigation is needed to conclusively state that *SETD2*-mutated lung adenocarcinoma has more favorable biology than non-mutated cancers.

This study has several other limitations. First, our sample size did not support the analysis of recurrence-free survival, which we were unable to stratified by individual stages. Stage I and II tumors generally follow similar treatment algorithms and are thus often grouped and analyzed as early-stage [22]; however, stratifying by individual stages may improve future studies. Analyzing the effect of the SETD2 mutation at each individual stage would provide more precise information about its relation to prognoses and outcomes. Further, analyzing the mutational profile at each stage of the tumor would add additional detail about molecular profiling. Additionally, our control group was initially derived from a convenience sample of 500 consecutive cases that presented in a more condensed timeframe than the SETD2-mutated cohort—the control group largely came from 2018 to 2020, while the *SETD2* group spanned a longer time period. As such, there may be differences in treatment patterns not captured by this study. For example, immunotherapy has been more frequently utilized in more recent treatment paradigms. This study’s time period also spans the implementation of the AJCC 8th edition for lung cancer stage classification [23]. Given our sample size and the relative rarity of *SETD2*-mutated tumors, we were unable to sample a more condensed time period and were unable to stratify analysis by treatment era. Future, larger studies with more modern cohorts would benefit from this stratification, with a specific focus on the response to immunotherapy. Future prospective studies would mitigate this limitation, and as Next-Generation Sequencing is now routine this is now feasible. Further, our study did not collect granular data on comorbidity, which may limit our ability to analyze RFS. Alternatively, utilizing a composite comorbidity score such as the Charlson–Deyo Comorbidity Index may be appropriate. Future studies may also benefit from a more granular collection of pathologic characteristics to more fully study the tumor biology related to the *SETD2* mutation status. The inclusion of lymphovascular invasion, spread through air spaces, or other pathologic variables may also elucidate the tumor biology of *SETD2*-mutated lung adenocarcinoma. Similarly, a promising area of investigation in *SETD2*-mutated cancer is into immune checkpoint expression, such as HAVCR2, CTLA-4, or TIGIT or biomarkers such as CDCP1 and AXL. Understanding the differential expression of these markers would further elucidate the etiology of favorable outcomes in *SETD2*-mutated lung cancer.

## 5. Conclusions

With the largest cohort of *SETD2*-mutated lung adenocarcinoma to date, we demonstrate that *SETD2* mutation is associated with a presentation at an earlier stage and trends towards an association with improved recurrence-free survival. We also demonstrate that *SETD2*-mutated tumors have a unique molecular profile compared to non-*SETD2* mutant tumors. This study is also hypothesis-generating—we demonstrate clinical associations and unique molecular profiles that may suggest underlying differences in tumor biology or behavior. This is an area of promising investigation and may lead to improved understandings that may facilitate new therapies or applications of existing therapies. Future larger and more granular study is warranted on both the outcomes and prognosis of patients with *SETD2*-mutated tumors as well as immunopathologic characteristics of *SETD2*-mutated lung cancer.

## Figures and Tables

**Table 1:** A Comparison of clinical characteristics in patients with and without *SETD2* mutation.

	Full Cohort		*SETD2*-Mutated		Control		*p* Value
Patient Characteristics	Median, N	IQR, %					
Age	68	[62–74]	68	[62–74]	68	[62–74]	0.92
BMI	26.5	[23.1–31.1]	27.1	[23.6–31.6]	26.2	[22.6–30.8]	0.39
Gender (M)	96	39.8%	22	32.8%	74	42.5%	0.19
Diabetes	52	21.6%	11	16.4%	41	19.9%	0.29
History of Tobacco Smoking	200	83.0%	53	79.1%	147	71.4%	0.34
COPD	95	39.4%	25	37.3%	70	34.0%	0.77
Asthma	49	20.3%	15	22.4%	34	16.5%	0.72
Asbestos	19	7.9%	5	7.5%	14	6.8%	0.61
Tumor Characteristics	Median, N	IQR, %					
T Stage							**<0.001**
T1	85	35.3%	37	55.2%	48	27.6%	
T2	44	18.3%	11	16.4%	33	19.0%	
T3	29	12.0%	8	11.9%	21	12.1%	
T4	40	16.6%	7	10.4%	33	19.0%	
TX	43	17.8%	4	6.0%	39	22.4%	
N Stage							**<0.001**
N0	108	44.8%	46	68.7%	62	35.6%	
N1	13	5.4%	4	6.0%	9	5.2%	
N2	50	20.7%	11	16.4%	39	22.4%	
N3	27	11.2%	2	3.0%	25	14.4%	
NX	43	17.8%	4	6.0%	39	22.4%	
M Stage							**<0.001**
M0	125	51.9%	20	29.9%	83	47.7%	
M1	85	35.3%	8	11.9%	67	38.5%	
MX	63	26.1%	39	58.2%	24	13.8%	
Clinical Stage							**<0.001**
I	68	28.2%	37	55.2%	31	17.8%	
II	30	12.4%	9	13.4%	21	12.1%	
III	51	21.2%	13	19.4%	38	21.8%	
IV	92	38.2%	8	11.9%	84	48.3%	
PD-L1							
Median Expression	2	IQR = [0–50]	1.5	[0–40]	2	[0–60]	0.45
Mean Expression	24.6	SD = 34.7	20.5	30.8	26.2	35.9	0.45
PD-L1-Positive (>10%)	99	41.1%	24	35.8%	75	43.1%	0.33
Gene Mutations	8	[6–12]	11	[9–16]	7	[5–11]	**<0.001**

*p* values less than 0.05 are bolded.

**Table 2:** Comparison of co-occurring mutations in *SETD2*-mutated and non-mutated tumors.

	*SETD2*-Mutated		Non-*SETD2*-Mutated		
Gene	N	%	N	%	*p* Value
*TMB*	48	71.6%	46	26.4%	<0.001
*BRAF*	37	55.2%	113	64.9%	0.1
*KRAS*	32	47.8%	59	33.9%	0.03
*TP53*	31	46.3%	111	63.8%	0.01
*MET*	28	41.8%	119	68.4%	<0.001
*EGFR*	27	40.3%	128	73.6%	<0.001
*ERBB2*	25	37.3%	116	66.7%	<0.001
*RET*	15	22.4%	24	13.8%	0.08
*ATM*	13	19.4%	21	12.1%	0.1
*KMT2C*	13	19.4%	15	8.6%	0.02
*SLIT2*	12	17.9%	7	4.0%	0.001
*ARID1A*	10	14.9%	15	8.6%	0.11
*DNMT3A*	10	14.9%	12	6.9%	0.05
*NF1*	10	14.9%	13	7.5%	0.07
*NOTCH2*	10	14.9%	8	4.6%	0.01
*MED12*	9	13.4%	2	1.1%	<0.001
*NOTCH3*	9	13.4%	11	6.3%	0.07
*NTRK3*	9	13.4%	13	7.5%	0.12
*TSC2*	9	13.4%	6	3.4%	0.007
*ALK*	8	11.9%	12	6.9%	0.16
*BRCA2*	8	11.9%	9	5.2%	0.06
*NTRK1*	8	11.9%	3	1.7%	0.002
*STK11*	8	11.9%	21	12.1%	0.059
*BRCA1*	7	10.4%	7	4.0%	0.06
*ERBB4*	7	10.4%	10	5.7%	0.16
*KMT2D*	7	10.4%	0	0.0%	<0.001
*PIK3CA*	7	10.4%	12	6.9%	0.25
*SMAD4*	7	10.4%	11	6.3%	0.2
*TET2*	7	10.4%	7	4.0%	0.06
*TSHR*	7	10.4%	3	1.7%	0.006

**Table 3:** Analysis of recurrence-free survival in patients with and without *SETD2* mutation.

Unadjusted Survival	N (%)	*SETD2*-Mutated	Non-Mutated	*p* = 0.007
One-Year	189 (78.33%)	60 (89.5%)	129 (73.9%)	
Three-Year	132 (54.9%)	48 (71.7%)	85 (49.1%)	
Five-Year	115 (48%)	41 (61%)	75 (43.2%)	
**Unadjusted Univariate Hazard Ratios**	**HR**	**95% CI**		***p* Value**
*SETD2* Mutation	0.53	0.33	0.85	**0.008**
Age	1.16	0.98	1.38	*0.08*
Male	1.03	0.72	1.48	0.86
BMI	1.10	0.85	1.44	0.46
Diabetes	1.37	0.91	2.06	*0.13*
Smoking	1.27	0.78	2.11	0.33
COPD	1.40	0.98	2.00	*0.06*
Asthma	1.18	0.78	1.82	0.43
Asbestos	1.00	1.00	1.00	0.65
Advanced Stage	1.59	1.11	2.33	**0.01**
PD-L1	0.99	1.00	1.00	0.33
Total	0.99	0.96	1.03	0.76
**Adjusted Multivariate Cox Hazard Ratios**				
*SETD2* Mutation	0.71	0.43	1.19	*0.10*
Age	1.20	0.99	1.45	*0.06*
Diabetes	1.29	0.85	1.95	0.29
COPD	1.23	0.83	1.82	*0.09*
Advanced Stage	1.25	1.06	1.48	**0.02**

*p* values less than 0.05 are bolded. *p* values less than or equal to 0.1 are italicized.

## Data Availability

Dataset used for this manuscript is available upon request.

## Supplementary Materials

The following supporting information can be downloaded at https://www.mdpi.com/article/10.3390/cancers17213540/s1, Table S1: *SETD2* Mutation Types; Supplemental Methods: Next-Generation Sequencing (NGS).

## Abbreviations

SETD2	SET domain containing 2
NSCLC	Non-small cell lung cancer
COPD	Chronic obstructive pulmonary disease
RFS	Recurrence-free survival
IQR	Interquartile range
CI	Confidence interval
HR	Hazard ratio
