# Supplementary material for: Characterizing the Clinical and Molecular Profile of SETD2-Mutated Lung Adenocarcinoma
Source: Cancers (Basel). 2025 Nov 1;17(21):3540. doi: 10.3390/cancers17213540 (PMC12610740; doi:10.3390/cancers17213540)
Supplement: Supplementary file 1 [file cancers-17-03540-s001.zip › cancers-3934486-supplementary.pdf]

Supplemental Table S1: *SETD2* Mutation Types

SETD2 Mutation Type	N (93)	%
Missense	35	37.6%
Frameshift	27	29.0%
Nonsense	19	20.4%
Splice Site	8	8.6%
Frameshift & Nonsense	2	2.2%
Inframe Deletion or Insertion	2	2.2%

Supplemental Methods

Next-Generation Sequencing (NGS)

Specimens were formalin-fixed paraffin-embedded (FFPE) tissue or in cytologic media. Specimens were required to have a minimal estimated tumor cellularity of 10%, as determined by pathologist review of the corresponding microscopic slide. DNA was extracted from specimen and processed for sequencing per kit manufacturer instructions, and then sequenced on an Illumina instrument.

Genetic variants, namely single nucleotide variants (SNVs), insertion-deletions (indels), and copy number variants (CNVs, depending on panel), were determined from sequencing data by custom bioinformatics pipeline. Genetic variants were reviewed, visualized using the Integrative Genomics Viewer (igv.org), and classified as Disease-Associated (equivalent to Pathogenic), Probably Disease Associated (equivalent to Likely Pathogenic), Variant of Uncertain Significance, Likely Benign, or Benign. Genetic variant classification was based on review and synthesis of evidence from the research literature and publicly-accessible online databases for cancer and/or germline genomics (including COSMIC, cBioPortal, ClinVar, ExAC, gnomAD).

Solid v2. This panel assessed SNVs and indels in 153 genes: *ABL1, AKT1, AKT2, AKT3, ALK, APC, AR, ARAF, ARID1A, ARID2, ATM, ATRX, AURKA, BAP1, BRAF, BRCA1, BRCA2, BRIP, BTK, CCND1, CCND2, CCND3, CCNE1, CDH1, CDK4, CDK6, CDKN2A, CHEK2, CIC, CREBBP, CRKL, CSF1R, CTNNB1, DAXX, DDR2, DNMT3A, EGFR, EP300, EPHA3, ERBB2, ERBB3, ERBB4, ERCC2, ERG, ESR1, ESR2, EZH2, FBXW7, FGF3, FGFR1, FGFR2, FGFR3, FGFR4, FLT3, FUBP1, GATA3, GNA11, GNAQ, GNAS, HRAS, H3F3A, IDH1, IDH2, IGF1R, JAK1, JAK2, JAK3, KCNG1, KDM5A, KDM5C, KDM6A, KDR, KIT, KMT2C, KRAS, LRRK2, MAP2K1, MAP2K2, MAP2K4, MAPK1, MAPK3, MAX, MCL1, MDM2, MDM4, MED12, MEN1, MET, MITF, MLH1, MRE11A, MSH2, MSH6, MTOR, MYC, MYCN, NBN, NF1, NF2, NTRK1, NTRK2, NTRK3, NKX2-1, NOTCH1, NOTCH2, NOTCH3, NRAS, PAK1, PALB2, PBRM1, PDGFRA, PIK3CA, PIK3CB, PIK3R1, PTCH1, PTEN, PTPN11, RAB35, RAC1, RAD50, RAD51, RAD51B, RAD51C, RAD51D, RAF1, RB1, RET, RHOA, RNF43, SETD2, SF3B1, SLIT2, SMAD4, SMARCA4, SMO, SPOP, SRC, STAG2, STK11, SUFU, SUZ12, SYK, TET2, TGFBR2, TP53, TRAF7, TSC1, TSC2, TSHR, U2AF1, VHL, WT1, XRCC2*. Targeted genetic regions were enriched by amplicon method using the Agilent HaloPlex kit (Agilent, Santa Clara, CA), and sequenced on a HiSeq 2500 instrument (Illumina, San Diego, CA). Bioinformatic sequence alignment and variant calling were made with respect to the GRCh37(hg19) reference genome build.

Solid v2 (revised). This panel assessed SNVs and indels in 152 genes: *ABL1, AKT1, AKT2, AKT3, ALK, APC, AR, ARAF, ARID1A, ARID2, ATM, ATRX, AURKA, BAP1, BRAF, BRCA1, BRCA2, BRIP, BTK, CREBBP, CCND1, CCND2, CCND3, CCNE1, CDH1, CDK4, CDK6, CDKN2A, CHEK2, CIC, CRKL, CSF1R, CTNNB1, DAXX, DDR2, DNMT3A, EIF1AX, EGFR, EP300, EPHA3, ERBB2, ERBB3, ERBB4, ERCC2, ERG, ESR1, ESR2, EZH2, FBXW7, FGF3, FGFR1, FGFR2, FGFR3, FGFR4, FLT3, FUBP1, GATA3, GNA11, GNAQ, GNAS, HRAS, H3F3A, IDH1, IDH2, IGF1R, JAK1, JAK2, JAK3, KDM5A, KDM5C, KDM6A, KDR, KIT, KMT2C, KRAS, MAP2K1, MAP2K2, MAP2K4, MAPK1, MAPK3, MAX, MCL1, MDM2, MDM4, MED12, MEN1, MET, MITF, MLH1, MRE11A, MSH2, MSH6, MTOR, MYC, MYCN, NBN, NF1, NF2, NKRT1,*

NKRT2, NKRT3, NKX2-1, NOTCH1, NOTCH2, NOTCH3, NRAS, PAK1, PALB2, PBRM1, PDGFRA, PIK3CA, PIK3CB, PIK3R1, PTCH1, PTEN, PTPN11, RAB35, RAC1, RAD50, RAD51, RAD51B, RAD51C, RAD51D, RAF1, RB1, RET, RHOA, RNF43, SETD2, SF3B1, SLIT2, SMAD4, SMARCA4, SMO, SPOP, SRC, STAG2, STK11, SUFU, SUZ12, SYK, TET2, TGFBR2, TP53, TRAF7, TSC1, TSHR, TSC2, U2AF1, VHL, WT1, XRCC2. This panel also assessed for copy number gains in 29 genes: *AKT1, AKT3, ALK, AR, ARAF, BRAF, BRCA1, BTK, CREBBP, CHEK2, CTNNB1, DDR2, EGFR, ERBB2, ESR1, GNA11, GNAQ, HRAS, IDH1, IDH2, KDR, KIT, KRAS, MAP2K1, MET, NRAS, PDGFRA, PIK3CA, RET*. Targeted genetic regions were enriched by amplicon method using the Agilent HaloPlex kit (Agilent, Santa Clara, CA), and sequenced on a HiSeq 2500 instrument (Illumina, San Diego, CA). Bioinformatic sequence alignment and variant calling were made with respect to the GRCh37(hg19) reference genome build.

PennSeq™ Solid v1. This panel assessed SNVs and indels in 183 genes: *ABL1, AKT1, AKT2, AKT3, ALK, APC, AR, ARAF, ARID1A, ARID2, ATM, ATRX, AURKA, AXIN1, B2M, BAP1, BCL2, BRAF, BRCA1, BRCA2, BRIP1, BTK, CCND1, CCND2, CCND3, CCNE1, CDH1, CDK4, CDK6, CDKN2A, CDKN2B, CHEK2, CIC, CREBBP, CRKL, CSF1R, CTNNB1, DAXX, DDR2, DDX41, DICER1, DNMT3A, EGFR, EIF1AX, EP300, EPCAM, EPHA3, ERBB2, ERBB3, ERBB4, ERCC2, ERG, ESR1, ESR2, EZH2, FBXW7, FGF3, FGFR1, FGFR2, FGFR3, FGFR4, FLT3, FOXL2, FUBP1, GATA3, GNA11, GNAQ, GNAS, H3-3A, HNF1A, HRAS, IDH1, IDH2, IGF1R, IKZF1, JAK1, JAK2, JAK3, KDM5A, KDM5C, KDM6A, KDR, KIT, KMT2C, KMT2D, KRAS, MAP2K1, MAP2K2, MAP2K4, MAPK1, MAPK3, MAX, MCL1, MDM2, MDM4, MED12, MEN1, MET, MITF, MLH1, MLH3, MPL, MRE11, MSH2, MSH3, MSH6, MTOR, MUTYH, MYC, MYCN, NBN, NF1, NF2, NKX2-1, NOTCH1, NOTCH2, NOTCH3, NPM1, NRAS, NTRK1, NTRK2, NTRK3, PAK1, PALB2, PBRM1, PDGFRA, PIK3CA, PIK3CB, PIK3R1, PMS1, PMS2, POLD1, POLE, POT1, PPM1D, PRPF8, PTCH1, PTEN, PTPN11, RAB35, RAC1, RAD50, RAD51, RAD51B, RAD51C, RAD51D, RAF1, RB1, RET, RHOA, RNF43, ROS1, SDHA, SDHB, SDHC, SDHD, SETD2, SF3B1, SLIT2, SMAD2, SMAD4, SMARCA4, SMARCB1, SMO, SPOP, SRC, STAG2, STK11, SUFU, SUZ12, SYK, TERT, TET2, TGFBR2, TP53, TRAF7, TSC1, TSC2, TSHR, U2AF1, VHL, WT1, XRCC2*. This panel also assessed for copy number gains in 17 genes: *AKT1, AKT3, BRAF, CTNNB1, DDR2, EGFR, ERBB2, ESR1, HRAS, KDR, KIT, KRAS, MET, NRAS, PDGFRA, PIK3CA, RET*. Targeted genetic regions were enriched by hybrid capture using a customized xGen Lockdown Probes kit (Integrated DNA Technologies, Coralville, IA) curated by the Genomics Organization for Academic Laboratories (www.goalabs.org), and performed on a HiSeq 2500 or NovaSeq 6000 instrument (Illumina, San Diego, CA). Bioinformatic sequence alignment and variant calling were made with respect to the GRCh38(hg38) reference genome build.
